# Preprocedural transthoracic Doppler echocardiography to identify stenosis associated with increased coronary flow after revascularisation

**DOI:** 10.1038/s41598-022-05683-0

**Published:** 2022-01-31

**Authors:** Masao Yamaguchi, Masahiro Hoshino, Tomoyo Sugiyama, Yoshihisa Kanaji, Kai Nogami, Tatsuhiro Nagamine, Toru Misawa, Masahiro Hada, Makoto Araki, Rikuta Hamaya, Eisuke Usui, Tadashi Murai, Tetsumin Lee, Taishi Yonetsu, Tetsuo Sasano, Tsunekazu Kakuta

**Affiliations:** 1 Division of Cardiovascular Medicine, Tsuchiura Kyodo General Hospital, 4-1-1 Otsuno, Tsuchiura City, Ibaraki, 300-0028 Japan; 2 Department of Cardiovascular Medicine, Tokyo Medical and Dental University, Tokyo, Japan

**Keywords:** Physiology, Cardiovascular biology, Circulation, Cardiology, Cardiovascular biology, Interventional cardiology

## Abstract

The benefit of percutaneous coronary intervention (PCI) has been reported to be associated with functional stenosis severity defined by fractional flow reserve (FFR). This study aimed to investigate the predictive ability of preprocedural transthoracic Doppler echocardiography (TDE) for increased coronary flow. A total of 50 left anterior descending arteries (LAD) that underwent TDE examinations were analysed. Hyperaemic LAD diastolic peak velocity (hDPV) was used as a surrogate of volumetric coronary flow. The increase in coronary flow was evaluated by the metric of % hDPV-increase defined by 100× (post-PCI hDPV-pre-PCI hDPV)/pre-PCI hDPV. The two groups divided by the median value of % hDPV-increase were compared, and the determinants of a significant coronary flow increase defined as more than the median % hDPV-increase were explored. After PCI, FFR values improved in all cases. hDPV significantly increased from 53.0 to 76.0 mm/s (P < 0.01) and the median % hDPV-increase was 45%, while hDPV decreased in 10 patients. On multivariable analysis, pre-PCI FFR and hDPV were independent predictors of a significant coronary flow increase. Preprocedural TDE-derived hDPV provided significant improvement of identification of lesions that benefit from revascularisation with respect to significant coronary flow increase.

## Introduction

Fractional flow reserve (FFR) can be used as an invasive marker to identify epicardial lesions that may benefit from revascularisation. FFR has shown prognostic efficacy and a continuous and independent relationship with subsequent outcomes^1,2^. An increase in coronary flow to the ischaemic region is the most fundamental reason for revascularisation because the severity and extent of stress-induced myocardial ischaemia have been proposed to be the most important contributing factors of a better prognosis^3,4^. These findings suggest that the benefit of percutaneous coronary intervention (PCI) may be greater in patients with lesions showing lower FFR values^5^, wherein a greater increase in coronary flow might be expected. PCI that provides no benefit with respect to coronary flow increase is a questionable indication and may even harm patients by exposing them to procedure- and stent-related risks, including the high bleeding risk of dual antiplatelet therapy. However, limited data are available regarding the relationship between physiological indices, such as FFR, coronary flow reserve (CFR), the index of microcirculatory resistance (IMR), hyperaemic peak coronary flow velocity, and changes in volumetric coronary blood flow after PCI. Furthermore, there are currently no preprocedural non-invasive markers that show predictive efficacy for a significant coronary flow increase after PCI.

Stress transthoracic Doppler echocardiography (S-TDE) is a cost-effective modality that provides diagnostic and prognostic information on coronary flow velocity and coronary flow velocity reserve (CFVR) without the need for ionising radiation, radioactive tracers, gadolinium, or intravascular catheterisation^6^. The CFVR value obtained by S-TDE represents useful quantitative information on the functional status of coronary artery circulation^7,8^. However, few S-TDE studies have documented changes in hyperaemic diastolic peak velocity (hDPV) after elective PCI. Vasodilator S-TDE, when performed after PCI, is likely to overcome most of the limitations of the wire-based, invasive, approach for measuring intracoronary pressure, such as under- or over-estimation of CFR and FFR because of reactive resting hyperaemia and microvascular injury^7,9,10^. Therefore, the three-fold aim of the present study, using hDPV as a surrogate of volumetric coronary flow, was to (1) investigate the early changes in S-TDE-derived indices, such as hDPV and CFVR, in the left anterior descending coronary artery (LAD) after successful and uncomplicated PCI; (2) explore the determinants of the changes in LAD hDPV; and (3) assess whether preprocedural S-TDE-derived physiological indices can predict increased coronary flow after PCI, independent of FFR.

## Materials and methods

### Study design and patient population

This study prospectively included 57 patients with stable coronary artery disease who were scheduled to undergo elective PCI of de novo*,* single, functionally significant LAD lesions at a single tertiary-care centre between June 10, 2020 and September 30, 2020. All patients had anginal symptoms (Canadian Cardiovascular Society class 1–3) and de novo*,* functionally significant, proximal LAD lesions (FFR ≤ 0.80). Patients with acute coronary syndrome or angiographically visible collateral flow were excluded. Other exclusion criteria were the inability to provide consent, history of myocardial infarction in the LAD territory, coronary intervention or coronary artery bypass graft surgery, occluded target vessels, left main coronary artery disease, reduced systolic function (ejection fraction < 50%), chronic renal disease, congestive heart failure, atrial fibrillation, and contraindications to adenosine administration. Optimal medical therapy with high-dose statins, dual antiplatelets, and antihypertensives was initiated immediately after diagnostic catheterisation in all patients. According to the study protocol, no ad hoc PCI was performed in this study.

The present study was approved by an institutional ethics committee (reference #955/Tsuchiura Kyodo General Hospital; 9 June, 2020) and was conducted in compliance with the tenets of the Declaration of Helsinki for human studies. All patients provided written informed consent for the study and future data utilisation.

#### Invasive coronary angiography

Each patient initially underwent standard diagnostic coronary angiography via the radial artery using a 6F system to assess the coronary anatomy and the severity of functional stenosis. Quantitative coronary angiography (QCA) analyses were performed using a CMS-MEDIS system (Medis Medical Imaging Systems, Leiden, Netherlands). All patients received a bolus injection of heparin (5000 IU) before the procedure. Intracoronary bolus injections of nitroglycerin (0.2 mg) were administered at the start of the procedure and before functional measurements.

#### Physiological measurements

Physiological measurements were performed in the LAD using a Radi Analyzer Xpress instrument with a single 0.014-inch PressureWire™ (Abbott Vascular, St. Paul, MN, USA). The FFR, mean transit time, CFR, and IMR were determined using a RadiAnalyzer Xpress instrument with a pressure–temperature sensor-chipped wire (Abbott Vascular), as per previously described methods. The FFR value was calculated as the ratio of the mean distal coronary pressure to the mean aortic pressure during stable hyperaemia, which was induced by the intravenous administration of adenosine (140 μg/kg/min through a central vein). After calibration, the wire was advanced, and the intracoronary pressure distal to the coronary stenosis was measured. After FFR measurement, when the pressure sensor reached the tip of the guiding catheter during hyperaemia via a pull-back manoeuvre, a mean Pd–Pa pressure drift of ≤ 2 mmHg was confirmed and documented. The institutional standard protocol mandated repeat assessment if the pressure drift was > 2 mmHg. All patients were instructed to strictly refrain from ingesting caffeinated beverages for > 24 h before catheterisation. CFR was calculated as resting mean transit time (Tmn) divided by hyperemic Tmn using a thermodilution technique^11^. Maximal hyperemia was induced by a continuous infusion at a rate of 140 μg/kg per min until a steady state hyperemia.

#### Percutaneous coronary intervention

PCI was performed according to the latest guidelines^12^. All patients underwent coronary stent implantation (2nd or 3rd generation drug-eluting stent) with pre-dilatation. The type of stent was selected at the operator’s discretion, and the strategy was determined by the interventionist. To avoid aggressive stent expansion, online QCA was performed to determine the proper stent size. Successful and uncomplicated PCI was defined as residual stenosis of < 20%, Thrombolysis in Myocardial Infarction flow grade of 3, no side branch occlusion or distal embolisation, and no PCI-related myocardial infarction according to the fourth universal definition of myocardial infarction^13^.

#### Measurement of coronary flow velocity

**Figure 1:**
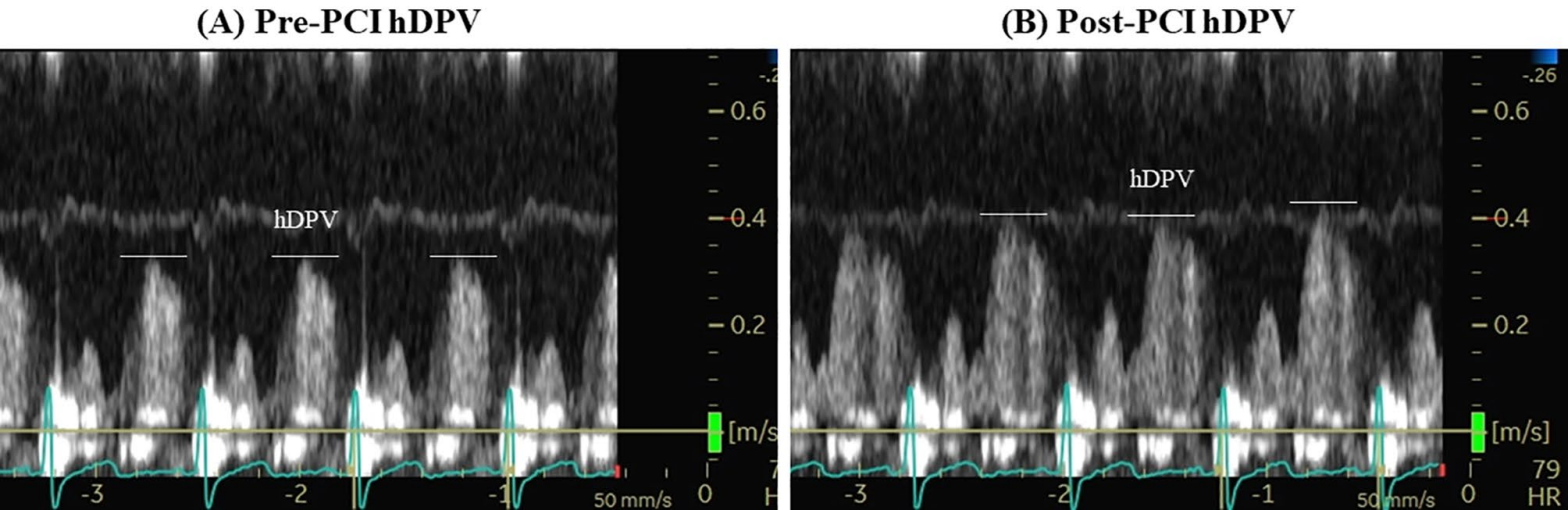
Representative images of coronary flow velocity measurements by transthoracic Doppler echocardiography. A representative case of a LAD lesion that underwent TDE examinations before and after successful PCI. (**A**) Pre-PCI hDPV, (**B**) post-PCI hDPV. *LAD* left anterior descending artery, *TDE* transthoracic Doppler echocardiography, *PCI* percutaneous coronary flow intervention, *hDPV* hyperaemic diastolic peak velocity.

Eligible patients underwent pre- (1 day before) and post-procedural (3 days after) LAD coronary flow assessments by S-TDE. Echocardiographic studies were performed according to the American Society of Echocardiography guidelines^14^ using a commercially available digital ultrasound system (GE Vivid E95; GE Vingmed Ultrasound, Horten, Norway) with a multifrequency transducer and second-harmonic technology. After the standard examination, coronary flow in the mid-distal portion of the LAD was visualised in a modified three-chamber view. For colour flow mapping, the velocity range was set as 16–24 cm/s. A sample volume (3–5 mm wide) was selected at the distal LAD to measure blood flow velocity. The peak diastolic coronary flow velocity was measured at basal conditions (bDPV) and during maximal hyperaemia (hDPV), which was induced by intravenous adenosine (140 μg/kg per min through a central vein). All data were digitally stored for offline review and measurements. Three optimal flow signal profiles at rest and during hyperaemia were obtained offline from the recorded data. The CFVR was calculated as the ratio of the hyperaemic peak diastolic flow velocity to the basal peak diastolic flow velocity^6^ using the software package of the ultrasound system. Coronary flow increase was evaluated by the metric of % hDPV-increase, defined as (post-PCI hDPV-pre-PCI hDPV)/pre-PCI hDPV × 100. Two groups categorised by the median value of % hDPV-increase were compared (significant increase group and non-significant increase group), and the determinants of a significant coronary flow increase, defined as an increase greater than the median % hDPV-increase, were investigated. The mean diastolic coronary flow velocity and velocity–time integral were measured at basal conditions (bDMV and bVTI, respectively) and during maximal hyperaemia (hDMV and hVTI, respectively). Both hDPV and hDMV showed significant predictive values in the univariate analysis. The AUCs of ROC analyses of hDPV and hDMV were 0.761 [95% CI 0.628–0.894] and 0.736 [95% CI 0.598–0.874], respectively (Supplemental Fig. S1). There were no significant differences in AUCs of ROC curves for these two different velocity measurements to predict significant coronary flow increase. Thus, hDPV, which showed a numerically larger AUC, was used for further analyses. Two experts who were blinded to the clinical data separately analysed all stored data at a 1-week interval and performed the analyses twice to evaluate the reproducibility of the S-TDE-derived data. Figure 1 shows a representative case of coronary flow velocity recording and measurement before and after LAD PCI.

### Statistical analysis

Statistical analyses were performed using R version 3.5.3. Categorical data are expressed as numbers and percentages and were compared using the chi-square or Fisher’s exact tests, as appropriate. The normality of the distributed values was assessed using Shapiro–Wilk statistics. Continuous variables are expressed as medians (25th–75th percentile) since all of the variables showed non-normal distributions and were compared using the Mann–Whitney U test. Associations were evaluated by analysing Spearman’s correlation for non-normally distributed data. Univariable linear regression analysis was performed to identify significant predictors of % hDPV-increase after PCI. Also, univariable logistic regression analyses were performed to predict a significant coronary flow increase. The associated variables with a P-value < 0.05 in the univariate analyses were entered in the multivariable model, and a forward stepwise regression method was used to fit the multivariable model. The Akaike information criterion was applied to test the model fitness to avoid over-fitting. Receiver operating characteristic (ROC) curve analysis was performed to assess the best cut-off values of pre-PCI FFR and pre-PCI hDPV to predict a significant coronary flow increase after PCI. The optimal cut-off value was calculated using the Youden index. A prediction model for significant coronary flow increase was constructed to determine the incremental discriminatory and reclassification performance of pre-PCI hDPV when it was added to the model including pre-PCI FFR; this involved the use of relative integrated discrimination improvement (IDI) and the category-free net reclassification index (NRI). A level of P < 0.05 was considered significant.

## Results

### Baseline patient characteristics and physiological findings

Of the 57 initially enrolled patients who underwent PCI for LAD lesions and S-TDE before and after PCI, five patients were excluded due to insufficient TDE data acquisition. Furthermore, one patient was excluded because they showed a type 4A myocardial infarction, and another patient withdrew their consent before completing the postprocedural S-TDE. Thus, the final analysis included 50 patients who underwent successful PCI and had complete TDE flow data.

**Figure 2:**
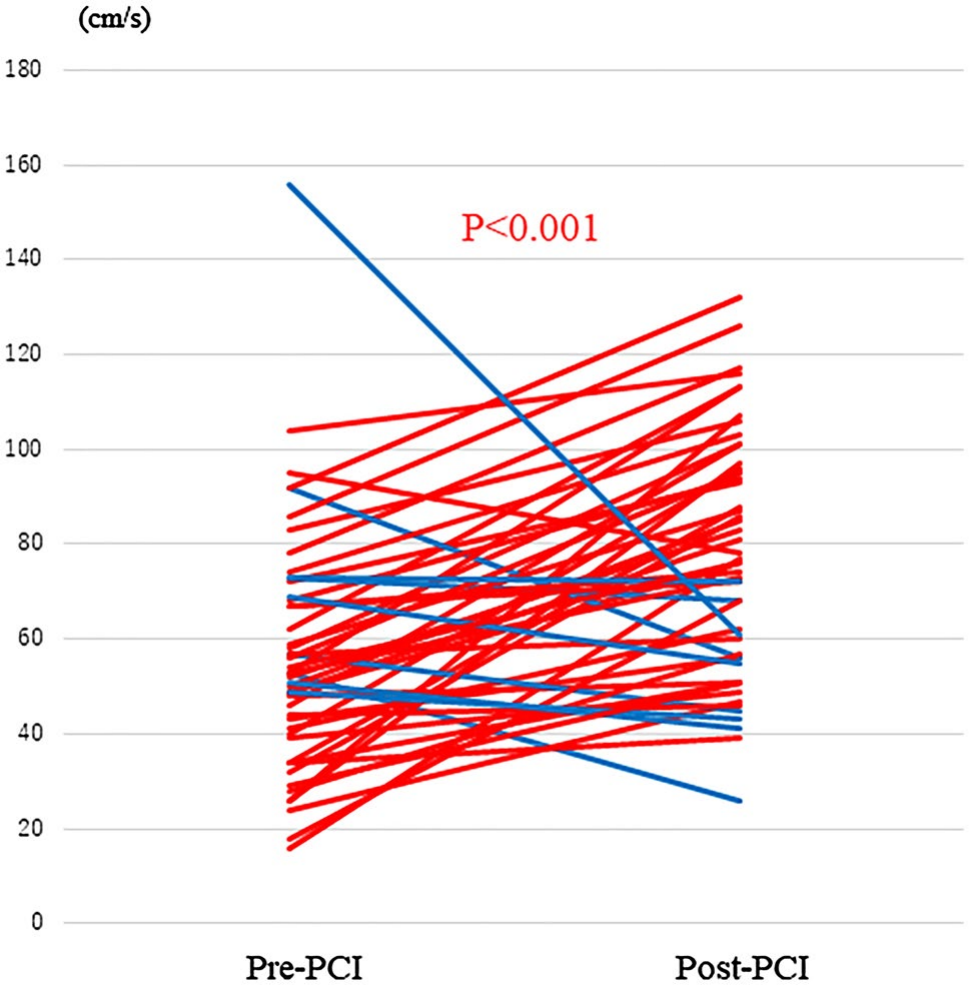
Serial changes in hDPV after PCI. hDPV significantly increased from 53.0 to 76.0 mm/s (P < 0.01), while hDPV decreased in 10 patients (20.0%). Red lines indicate cases with increased hDPV after PCI, and blue lines indicate cases with decreased hDPV after PCI. Abbreviations as in Fig. 1.

Table 1 summarises the baseline characteristics and angiographic and physiological data of 50 patients in the two groups divided by the median value of % hDPV-increase after PCI. The median % hDPV-increase was 45%. The FFR and pre-PCI hDPV were significantly lower in the significant coronary flow increase group (FFR: 0.65 vs 0.73, P = 0.005; pre-PCI hDPV: 46.0 vs 67.0 cm/s, P = 0.001). After successful PCI, hDPV significantly increased from 53.0 (39.8–69.8) cm/s to 76.0 (55.8–96.3) cm/s; however, 10 patients (20.0%) showed decreased hDPV despite of an increase in FFR (Fig. 2).

**Table 1:** Baseline patient characteristics.

	All patients (N = 50)	Significant increase group (N = 25)	Non-significant increase group (N = 25)	P-value
Age, years	71.0 (63.0–76.0)	71.0 (60.5–76.0)	72.0 (64.5–76.3)	0.528
Male, n (%)	39 (78)	20 (80)	19 (76)	0.732
Hypertension, n (%)	38 (76)	20 (80)	19 (76)	0.732
Diabetes mellitus, n (%)	21 (42)	11 (44)	10 (40)	0.775
Dyslipidemia, n (%)	24 (48)	12 (48)	12 (48)	1.000
Smoking, n (%)	25 (50)	12 (48)	13 (52)	0.777
eGFR, ml/min/1.73 m^2^	68.0 (52.5–77.2)	66.5 (52.4–79.5)	68.0 (47.3–77.2)	0.946
LDL-chol, mg/dl	77.5 (61.0–104.3)	73.0 (60.5–95.0)	83.0 (66.5–116.5)	0.277
NT-pro BNP, pg/ml	145.5 (67.8–479.8)	103.5 (50.8–395.3)	153.5 (117.3–637)	0.139
hs-TnI, ng/ml	0.008 (0.003–0.016)	0.008 (0.002–0.018)	0.009 (0.003–0.012)	0.761
HbA1c, %	6.2 (5.8–6.8)	6.2 (5.8–7.3)	6.0 (5.8–6.5)	0.330
**Medication (admission)**				
Statin, n (%)	47 (94)	23 (92)	24 (96)	0.552
ACEi or ARB, n (%)	29 (58)	13 (52)	16 (64)	0.390
βblocker, n (%)	28 (56)	16 (64)	12 (48)	0.322
**QCA analysis**				
QCA MLD	1.1 (0.9–1.4)	1.1 (0.8–1.5)	1.1 (0.9–1.4)	1.000
QCA RD	2.5 (2.2–3.0)	2.9 (2.4–3.5)	2.3 (2.1–2.7)	0.005
QCA %stenosis	56.4 (48.9–66.4)	57.8 (40.5–73.3)	55.6 (46.6–65.6)	0.089
QCA lesion length	22.4 (15.8–30.8)	25.8 (18.0–31.0)	20.0 (15.6–30.8)	0.172
**Baseline physiological indices**				
FFR	0.71 (0.63–0.76)	0.65 (0.54–0.73)	0.73 (0.68–0.77)	0.005
CFR	1.96 (1.36–3.13)	1.67 (1.18–2.66)	2.57 (1.66–3.54)	0.029
CFVR	1.96 (1.61–2.32)	1.90 (1.33–2.18)	2.00 (1.72–2.46)	0.079
IMR	24.4 (17.6–33.8)	22.0 (17.6–36.9)	25.4 (16.4–32.1)	0.969
bTmn, s	0.92 (0.66–1.45)	0.81 (0.58–1.48)	0.95 (0.68–1.44)	0.652
hTmn, s	0.45 (0.30–0.65)	0.53 (0.28–0.90)	0.43 (0.29–0.55)	0.099
bDPV, cm/s	28.5 (19.0–36.3)	29.0 (18.0–32.0)	28.0 (23.5–38.0)	0.164
hDPV, cm/s	53.0 (39.8–69.8)	46.0 (28.5–57.0)	67.0 (50.0–73.5)	0.001
bDMV, cm/s	20.0 (15.0–27.3)	19.0 (14.0–25.0)	21.0 (16.5–28.0)	0.203
hDMV, cm/s	41.0 (21.1–51.8)	37.0 (22.0–44.0)	47.0 (36.0–59.5)	0.004
bVTI, cm	10.4 (9.0–15.9)	10.5 (9.34–17.9)	9.8 (8.3–15.6)	0.882
hVTI, cm	19.2 (13.5–25.3)	16.6 (11.4–23.8)	21.7 (15.4–29.1)	0.073
**Baseline echocardiography parameters**				
LVDd, mm	46.0 (41.3–51.0)	46.0 (42.5–51.0)	45.0 (41.0–51.0)	0.702
VS, mm	11.0 (10.0–12.0)	11.0 (10.0–12.0)	11.0 (9.0–13.0)	0.899
PW, mm	11.0 (10.0–12.0)	11.0 (10.0–13.0)	11.0 (10.0–12.0)	0.549
LVEF, %	64.5 (60.0–68.3)	65.0 (63.0 -70.0)	62.0 (58.0–68.0)	0.120
E/A ratio	0.8 (0.6–0.9)	0.8 (0.7–1.0)	0.7 (0.6–0.9)	0.359
E/average e’	11.0 (9.0–15.8)	11.0 (8.0–12.5)	12.0 (9.0–18.0)	0.125
**Post-PCI physiological indices**				
FFR	0.85 (0.82–0.87)	0.84 (0.81–0.87)	0.85 (0.83–0.88)	0.212
CFR	2.71 (2.00–3.75)	2.89 (1.69–4.21)	2.60 (2.05–3.66)	0.323
CFVR	2.57 (2.14–3.30)	2.92 (2.42–3.49)	2.37 (1.84–2.78)	0.012
IMR	19.22 (15.13–23.96)	19.44 (11.80–23.07)	19.00 (16.38–31.17)	0.101
bTmn, s	0.86 (0.50–1.38)	0.80 (0.38–1.20)	0.92 (0.58–1.45)	0.261
hTmn, s	0.29 (0.22–0.38)	0.30 (0.18–0.37)	0.28 (0.24–0.49)	0.123
bDPV, cm/s	30.0 (23.8–35.0)	30.0 (22.5–34.5)	30.0 (23.5–35.0)	0.962
hDPV, cm/s	76.0 (55.8–96.3)	87.0 (72.0–104.0)	61.0 (47.5–77.0)	0.001
bDMV, cm/s	22.0 (17.0–26.0)	22.0 (16.0–25.0)	22.0 (17.5–26.5)	0.884
hDMV, cm/s	57.0 (38.0–70.3)	62.0 (52.0–80.0)	45.0 (35.5–63.5)	0.005
bVTI, cm	10.7 (7.5–13.5)	11.9 (9.1–15.7)	9.2 (7.0–11.9)	0.020
hVTI, cm	25.2 (17.2–34.4)	31.1 (24.4–40.3)	19.9 (12.9–25.2)	< 0.001
**Post-PCI echocardiography parameters**				
LVDd, mm	46.0 (42.0–50.0)	47.0 (43.5–50.5)	45.0 (42.0–49.0)	0.549
VS, mm	11.0 (10.0–12.0)	11.0 (10.0–12.0)	11.0 (9.0–12.0)	0.657
PW, mm	11.0 (10.0–12.0)	11.0 (10.0–13.0)	10.0 (10.0–12.0)	0.398
LVEF, %	65.0 (62.0–69.0)	65.0 (63.0 -71.5)	64.5 (60.0–68.0)	0.133
E/A ratio	0.8 (0.7–0.9)	0.8 (0.7–1.0)	0.7 (0.6–0.9)	0.398
E/average e’	11.0 (9.0–15.0)	11.0 (9.0–12.0)	12.0 (10.0–20.0)	0.132

Data are presented as n (%), mean SD, or median (interquartile range).

*A* peak inflow velocity during late diastole, *ACE-I* angiotensin-converting enzyme inhibitor, *ARB* angiotensin receptor blocker, *averaged e*′, average of septal e′ and lateral e′, *b* basal, *CFR* coronary flow reserve, *CFVR* coronary flow velocity reserve, *CRP* C-reactive protein, *DMV* diastolic mean velocity, *E* peak inflow velocity during early diastole, *e*′ mitral annular velocity during early diastole on the septal or lateral side, *h* hyperemic, *DPV* diastolic peak velocity, *DS* diameter stenosis, *eGFR* estimated glomerular filtration rate, *FFR* fractional flow reserve, *h* hyperemic, *HDL* high-density lipoprotein, *hs-TnI* high sense troponin I, *IMR* index of microcirculatory resistance, *LDL* low-density lipoprotein, *LV* left ventricular, *LVEF* left ventricular ejection fraction, *MLD* minimum lumen diameter, *%stenosis* percent diameter stenosis, *QCA* quantitative coronary angiography, *RD* reference lumen diameter, *Tmn* transit time, *VS* ventricular septal thickness, *VTI* velocity–time integral, *PW* posterior wall thickness.

### Predictors of a significant coronary flow increase and determinants of a change in hDPV after PCI

**Figure 3:**
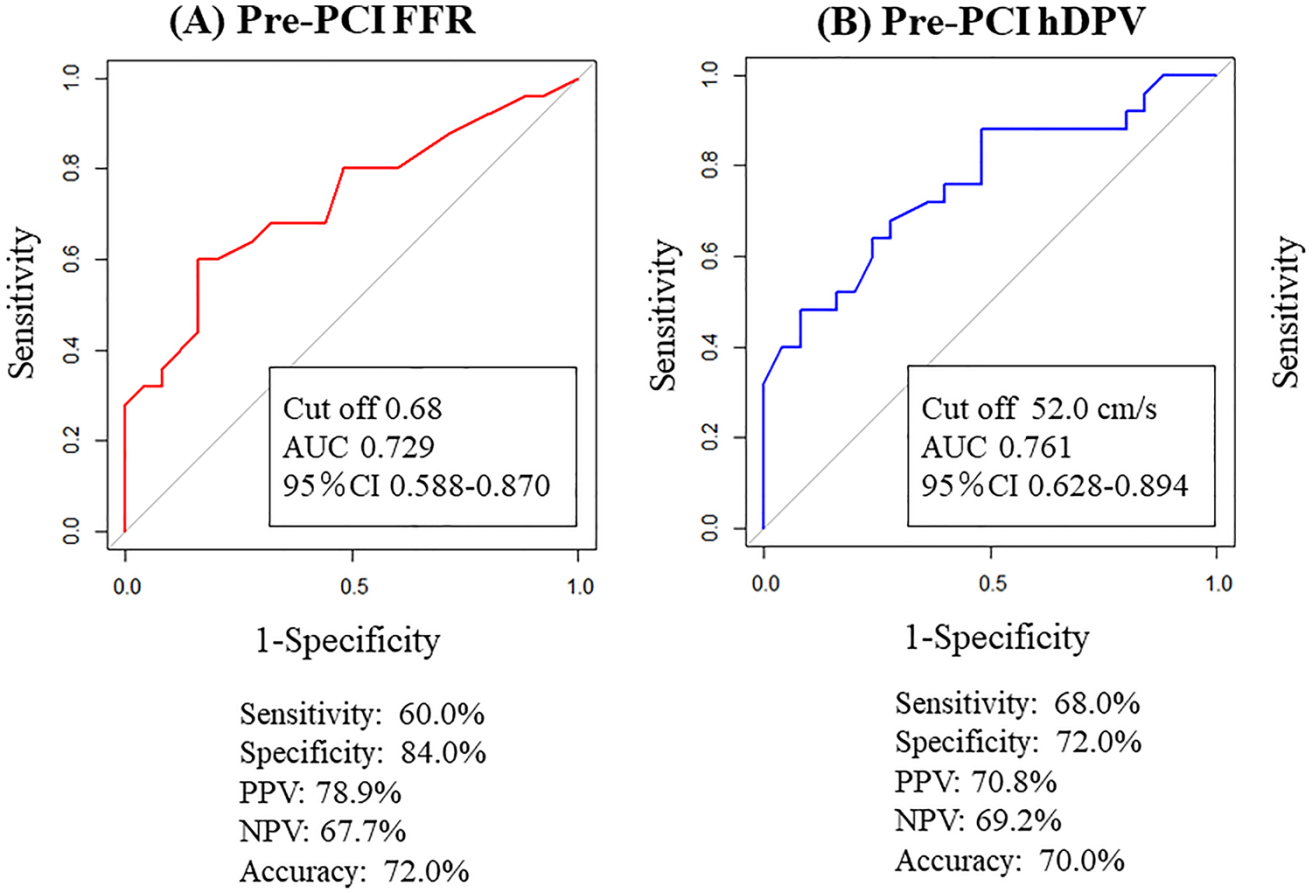
ROC analyses to determine the optimal cut-off values to predict a significant coronary flow increase. (**A**) ROC analysis of pre-PCI FFR to predict a significant coronary flow increase. (**B**) ROC analysis of pre-PCI hDPV to predict a significant coronary flow increase. *FFR* fractional flow reserve, *ROC* receiver operating characteristic; other abbreviations as in Fig. 1.

Predictors of a significant coronary flow increase were assessed using univariate and multivariate logistic regression analyses (Table 2). The pre-PCI FFR and pre-PCI hDPV were independently associated with a significant coronary flow increase. The results of univariable and multivariable linear regression analyses for predicting % hDPV-increase after PCI are shown in Table 3. Pre-PCI FFR and pre-PCI hDPV were independently and significantly associated with a % hDPV-increase after PCI. ROC analyses revealed the best cut-off values of pre-PCI FFR and pre-PCI hDPV for a significant coronary flow increase as follows: FFR = 0.68 (AUC: 0.729, 95% confidence interval [CI] 0.558–0.870) and hDPV = 52.0 cm/s (AUC, 0.761; 95% CI 0.628–0.894) (Fig. 3A,B). Table 4 shows that adding pre-PCI hDPV to the FFR model can increase predictability for a significant coronary flow increase (NRI: 0.560, 95% CI 0.044–1.075, P = 0.033 and IDI: 0.119, 95% CI 0.028–0.210, P = 0.009).

**Table 2:** Univariate and multivariate analysis predicting a significant coronary flow increase.

	Univariate logistic regression					Multivariate logistic regression				
	OR	95% CI			P-value	OR	95% CI			P-value
FFR	1.00E−05	7.24E−10	–	0.016	< 0.001	1.68E−05	7.32E−11	–	0.232	0.022
CFR	0.626	0.366	–	0.098	0.041					
CFVR	0.278	0.076	–	0.080	0.016					
IMR	1.016	0.981	–	1.059	0.383					
Hyperemic Tmn	9.758	1.470	–	129.009	0.015					
hDPV	0.951	0.914	–	0.981	< 0.001	0.961	0.919	–	0.997	0.031
hDMV	0.948	0.909	–	0.989	0.004					
QCA RD	3.940	1.573	–	12.871	0.002	2.667	0.998	–	8.819	0.051
QCA %stenosis	1.044	0.993	–	1.103	0.092					

*CFR* coronary flow reserve, *CFVR* coronary flow velocity reserve, *FFR* fractional flow reserve, *hDMV* hyperemic diastolic mean velocity, *hDPV* hyperemic diastolic peak velocity, *IMR* index of microcirculatory resistance, *%stenosis* percent diameter stenosis, *QCA* quantitative coronary angiography, *RD* reference lumen diameter, *Tmn* transit time.

**Table 3:** Univariate and multivariate linear analysis of factors predicting %hDPV-increase.

	Univariate					Multivariate				
	β	95% CI			P-value	β	95% CI			P-value
FFR	− 0.683	− 8.154	–	− 4.294	< 0.001	− 0.476	− 6.182	–	− 2.486	< 0.001
CFR	− 0.439	− 0.468	–	− 0.120	0.001	− 0.15	− 0.231	–	0.03	0.128
CFVR	− 0.391	− 1.128	–	− 0.363	< 0.001					
IMR	0.194	− 0.005	–	0.026	0.178					
Hyperemic Tmn	0.516	0.640	–	1.829	< 0.001					
hDPV	− 0.615	− 0.030	–	− 0.014	< 0.001	− 0.414	− 0.022	–	− 0.008	< 0.001
QCA MLD	− 0.211	− 1.056	–	0.154	0.140					
QCA RD	0.370	0.118	–	0.754	0.008					
QCA %stenosis	0.193	− 0.007	–	0.038	0.178					

*CFR* coronary flow reserve, *CFVR* coronary flow velocity reserve, *FFR* fractional flow reserve, *hDPV* hyperemic diastolic peak velocity, *IMR* index of microcirculatory resistance, *MLD* minimum lumen diameter, *%stenosis* percent diameter stenosis, *QCA* quantitative coronary angiography, *RD* reference lumen diameter, *Tmn* transit time.

**Table 4:** Prediction model for a significant coronary flow increase.

	C-statistics	P-value	IDI	P-value2	NRI	P-value3
FFR	0.728	–	Reference	–	Reference	–
FFR + hDPV	0.830	0.096	0.119	0.009	0.560	0.033

*IDI* integrated discrimination improvement, *FFR* fractional flow reserve, *hDPV* hyperemic diastolic peak velocity, *NRI* net reclassification improvement.

**Figure 4:**
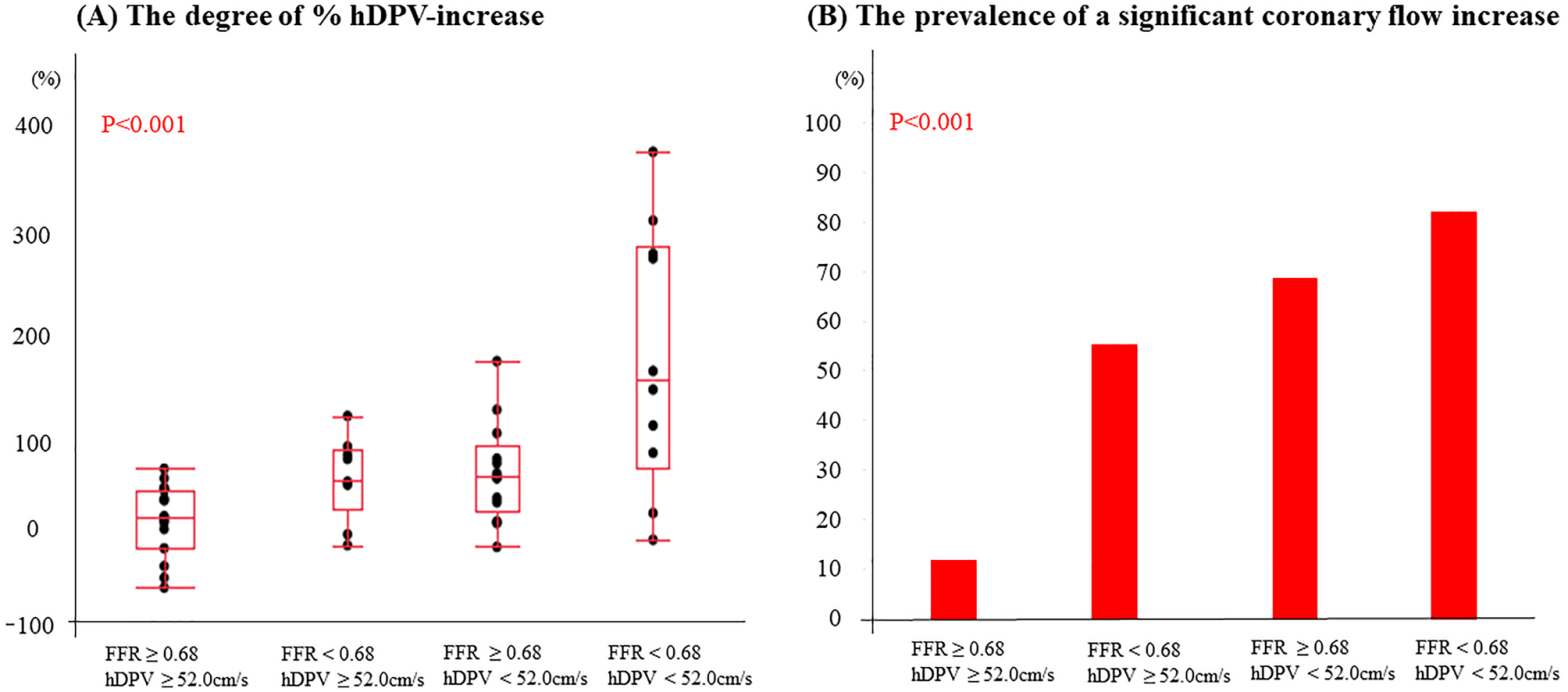
The degree of % hDPV-increase and the prevalence of a significant coronary flow increase in four groups stratified by the best cut-off values of pre-PCI FFR and pre-PCI hDPV (pre-PCI FFR = 0.68 and pre-PCI hDPV = 52.0 cm/s). (**A**) The degree of % hDPV-increase was significantly different between the four groups. (**B**) The prevalence of a significant coronary flow increase was significantly different between the four groups. Abbreviations as in Figs. 1 and 3.

When patients were divided into four groups based on the best cut-off values of FFR and pre-PCI hDPV (0.68 and 52.0 cm/s, respectively), the degree of % hDPV-increase and the prevalence of a significant coronary flow increase were significantly different between these four groups (Fig. 4A,B).

#### S-TDE measurement reproducibility

The inter- and intra-observer agreements for the identification of impaired CFVR (≤ 2.0) were 93% and 96%, respectively. The mean difference in CFVR values between the two observers was 6.8%.

## Discussion

The current study investigated the predictive ability of preprocedural TDE for increased coronary flow after elective PCI. The essential findings were as follows: (1) PCI increased hyperaemic coronary flow, documented by S-TDE-derived hDPV, in 80% of the study patients, while FFR improvement was observed in all patients; (2) the median coronary flow velocity increase was 45%; (3) pre-PCI FFR, pre-PCI CFR, QCA reference diameter, and pre-PCI hDPV were associated with a significant coronary flow increase; and (4) pre-PCI hDPV predicted a significant coronary flow increase independent of the pre-PCI FFR value. To the best of our knowledge, the present study is the first to demonstrate that non-invasive S-TDE examination can predict a significant coronary flow increase after PCI for functionally significant LAD lesions.

### Rationale of revascularisation

The aim of revascularisation by PCI is to increase coronary blood flow in an ischaemic region by modifying the epicardial lesion. A previous study using positron emission tomography (PET) showed a significant increase in regional stress myocardial blood flow (MBF) and myocardial flow reserve in the region with revascularisation^15^. However, limited data are available on the prediction of increased coronary flow after PCI or even on the relationship between baseline patient data, including those of functional lesion assessment, and coronary flow increase.

The FFR has rapidly gained consensus as the gold standard for representing induced regional ischaemia due to epicardial coronary artery stenosis for revascularisation decision-making in patients with stable coronary artery disease. The FFR has also been demonstrated to show a continuous and independent relationship with subsequent outcomes in patients with stable coronary artery disease (CAD)^2^. Based on these studies, a physiological rationale is increasingly required before decision-making for revascularisation in patients with chronic coronary syndrome. In addition, considering that the existence, extent, and severity of induced myocardial ischaemia have been proposed to be the most important contributing factors for a better prognosis^3,4^, coronary intervention without a corresponding increase in coronary flow or a reduction in ischaemia would not be expected to improve patient outcomes. Indeed, this could even be harmful due to the exposure of patients to procedure-related, stent-related, and high bleeding risks owing to dual antiplatelet therapy or a combination of anticoagulation therapies. Therefore, reliable and widely available methods are necessary to identify or predict a significant coronary flow increase after PCI, preferably by non-invasive pre-PCI testing.

### Determinants of coronary flow increase after PCI

Recently, Driessen et al. and Knaapen et al. reported that successful coronary revascularisation has a significant and positive impact on absolute myocardial perfusion as assessed by serial PET. Notably, the improvement in the FFR after PCI was directly related to the increase in hyperaemic MBF^16^. Kanaji et al., using cardiac magnetic resonance imaging, showed that FFR-guided PCI was associated with increased absolute hyperaemic coronary sinus flow and that increased flow was associated with pre-PCI FFR values^5^. In the present study, we demonstrated that using S-TDE, pre-PCI S-TDE-derived hDPV had a powerful ability for predicting coronary flow increase by multiple linear regression analysis independent of pre-PCI FFR values. Furthermore, we defined a significant coronary flow increase after PCI as an increase greater than the median improvement in hDPV (45%); this value was in line with that reported in a previous PET study, which showed an average increase of 46% in absolute coronary flow after PCI^17^. A significant coronary flow increaser could also be predicted independent of pre-PCI FFR values by multivariate analysis.

### Potential clinical implication of pre-PCI S-TDE examination

Our results indicated that the pre-PCI FFR is one of the most important determinants of increased coronary flow. However, ROC analysis showed that the best cut-off value of pre-PCI FFR for predicting a significant coronary flow increase was 0.68. This finding supports that of the prior study by Nijjer et al.^18^, in which the pre-PCI FFR predicted an increase in coronary flow velocity after PCI; the greatest increments in hyperaemic flow velocity were seen in territories where the pre-PCI FFR was ≤ 0.70. The present study strongly suggests that in addition to pre-PCI FFR values obtained invasively at the catheterisation laboratory, the non-invasive pre-PCI S-TDE-derived metric of hDPV could yield incremental predictive information for coronary flow increase after PCI. These findings support our decision-making regarding revascularisation, although further investigations are required.

### Limitations

This study has several limitations, which should be considered when interpreting the results. First, the present study evaluated only 50 patients for the final analysis and carried the inherent limitations due to its small sample size, single-centre study, and observational nature; therefore, precluded extensive subgroups analyses and a selection bias can not be canceled. Second, in the Doppler echocardiographic examination of LAD coronary flow, pre- and post-PCI LAD flow data were comparable only at identical measurement positions and constant vessel diameters under similar haemodynamics. These issues were carefully addressed by ensuring the exact positioning of the echo probe, performing functional assessments, and maintaining the specified time frame for examination (around 10 am). Nevertheless, we cannot exclude the possibility of a change in the diameter of the LAD after non-LAD PCI or a difference in the functional measurement position. Third, the absolute coronary flow volume was not assessed in this study. The limited coronary flow volume in the ischaemic region depends on the downstream myocardial mass subtended by the lesion, and the impact of PCI on the coronary flow of the LAD might be affected by restoration of coronary flow volume following revascularisation. However, the main strength of the present study was the paired comparison performed for serial TDE examinations of the proximal LAD lesions for each patient, which might at least partially alleviate this limitation. Fourth, coronary flow may serially change after PCI, and our results are based on one time window of 3 days after the procedure. Further studies are needed to serially quantify the changes in coronary flow if the S-TDE data obtained in the present study are shown to be able to predict flow characteristics at a later follow-up date. Fifth, no prognostic data was provided in the present study. Sixth, the post-PCI TDE measurement was performed at more than 24 h after PCI, which may have influenced on the results. Subsequent mid- and long-term changes in coronary flow should be further studied. Seventh, approximately 10% of patients were not adequately evaluated by echocardiography, which might have resulted in a certain bias for the results. Finally and most importantly, future studies should test whether changes in coronary flow in ischaemic regions after PCI could provide prognostic information.

## Conclusions

The noninvasively obtained pre-PCI S-TDE-derived hDPV could yield incremental predictive information over pre-PCI FFR values for a significant increase in coronary flow after PCI, which might support our decision to perform revascularisation.

## Supplementary Information

Supplementary Legends.

Supplementary Figure S1.
