# Supplementary material for: Preprocedural transthoracic Doppler echocardiography to identify stenosis associated with increased coronary flow after revascularisation
Source: Sci Rep. 2022 Jan 31;12:1667. doi: 10.1038/s41598-022-05683-0 (PMC8803832; doi:10.1038/s41598-022-05683-0)
Supplement: Supplementary file 1 — Supplementary Legends. [file 41598_2022_5683_MOESM1_ESM.docx]

**Preprocedural Transthoracic Doppler Echocardiography to Identify Stenosis Associated With Increased Coronary Flow After Revascularisation**

Masao Yamaguchi MD^1^; Masahiro Hoshino MD^1^; Tomoyo Sugiyama MD, PhD^1^; Yoshihisa Kanaji MD, PhD^1^; Kai Nogami MD^1^; Tatsuhiro Nagamine MD^1^; Toru Misawa MD^1^; Masahiro Hada MD^1^; Makoto Araki MD^1^; Rikuta Hamaya MD^1^; Eisuke Usui MD, PhD ^1^; Tadashi Murai MD, PhD^1^; Tetsumin Lee MD PhD^1^; Taishi Yonetsu MD^2^; Tetsuo Sasano MD, PhD^2^; and Tsunekazu Kakuta MD, PhD^1^

^1^Division of Cardiovascular Medicine, Tsuchiura Kyodo General Hospital, Ibaraki, Japan

^2^Department of Cardiovascular Medicine, Tokyo Medical and Dental University, Tokyo, Japan

Supplemental figure 1 Legend

Comparison of AUCs derived from ROC curves to predict a significant coronary flow increase.

hDPV, hyperaemic diastolic peak velocity; and hDMV hyperaemic diastolic mean velocity.
