# Supplementary figures and images for: Preprocedural transthoracic Doppler echocardiography to identify stenosis associated with increased coronary flow after revascularisation
Source: Sci Rep. 2022 Jan 31;12:1667. doi: 10.1038/s41598-022-05683-0 (PMC8803832; doi:10.1038/s41598-022-05683-0)

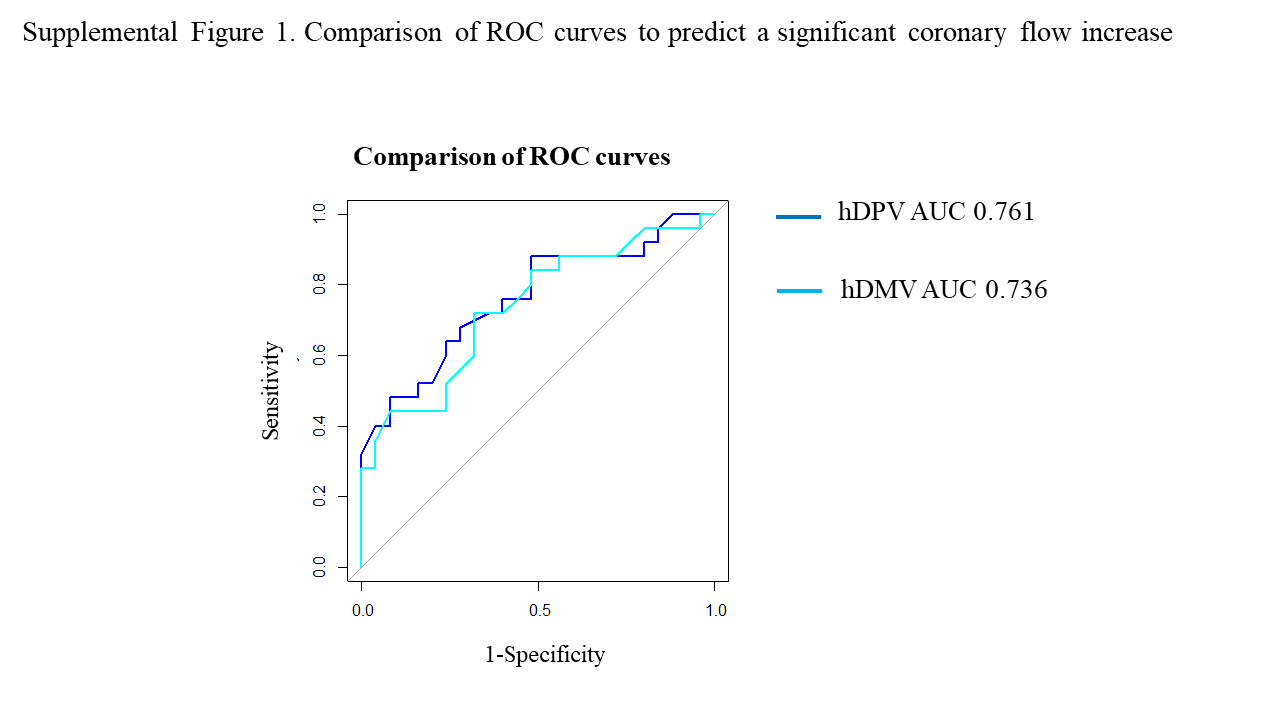

Supplement: Supplementary file 2 — Supplementary Figure S1. [file 41598_2022_5683_MOESM2_ESM.tif]
